# Psycholinguistic changes in the communication of adolescent users in a suicidal ideation online community during the COVID-19 pandemic

**DOI:** 10.1007/s00787-022-02067-7

**Published:** 2022-08-26

**Authors:** Johannes Feldhege, Markus Wolf, Markus Moessner, Stephanie Bauer

**Affiliations:** 1 Center for Psychotherapy Research, Heidelberg University Hospital, Bergheimer Strasse 54, 69115 Heidelberg, Germany; 2 Department of Psychology, University of Zurich, Zurich, Switzerland

**Keywords:** Suicidal ideation, Suicide attempt, Adolescents, Social media, Online self-help community, Topic modelling, Language style

## Abstract

Since the outbreak of the COVID-19 pandemic, increases in suicidal ideation and suicide attempts in adolescents have been registered. Many adolescents experiencing suicidal ideation turn to online communities for social support. In this retrospective observational study, we investigated the communication—language style, contents and user activity—in 7975 unique posts and 51,119 comments by *N* = 2862 active adolescent users in a large suicidal ideation support community (SISC) on the social media website reddit.com in the onset period of the COVID-19 pandemic. We found significant relative changes in language style markers for hopelessness such as negative emotion words (+ 10.00%) and positive emotion words (− 3.45%) as well as for social disengagement such as social references (− 8.63%) and 2nd person pronouns (− 33.97%) since the outbreak of the pandemic. Using topic modeling with Latent Dirichlet Allocation (LDA), we identified significant changes in content for the topics Hopelessness (+ 23.98%), Suicide Methods (+ 17.11%), Social Support (− 14.91%), and Reaching Out to users (− 28.97%). Changes in user activity point to an increased expression of mental health issues and decreased engagement with other users. The results indicate a potential shift in communication patterns with more adolescent users expressing their suicidal ideation rather than relating with or supporting other users during the COVID-19 pandemic.

**Supplementary Information:**

The online version contains supplementary material available at 10.1007/s00787-022-02067-7.

## Introduction

Suicide is one of the leading causes of death among adolescents around the world [1]. Adolescents are at risk for suicidal ideation with a 12 month prevalence at 14.2% [2]. While suicide attempts by adolescents may occur without prior suicidal ideation, the risk of a future suicide attempt is greatly increased by prior suicidal ideation [3].

The COVID-19 pandemic had a major psychological impact on distress, anxiety, depression as well as suicidal ideation and behaviors [4]. It was suggested that the COVID-19 pandemic could lead to a pandemic of suicides [5]. A meta-analysis across 54 studies established evidence for a rise in the rate of suicide attempts and the level of suicidal ideation during the COVID-19 pandemic [6]. An important moderator of suicide ideation in the meta-analysis was younger age. It is therefore not surprising that adolescents admitted to pediatric hospitals showed higher numbers of suicide attempts and higher levels of suicidal ideation in in the first months of the COVID-19 pandemic compared to the same timeframe in 2019 [7, 8].

To our knowledge, factors that could have led to these increases in suicidal behaviors in adolescents during the COVID-19 pandemic have not been identified yet, however the existing research on risk factors for suicidal behaviors can give some clues on the influence of the pandemic. Factors associated with the COVID-19 pandemic such as physical isolation due to lockdowns, quarantine or during school closures can contribute to social isolation and loneliness in adolescents [9]. This can lead to a rise in mental health issues, known risk factors for suicidal ideation and behaviors [10, 11]. Adolescents may increase their use of the internet and social media to stay in contact with their peers or to get help and support for mental health issues and suicidal ideation while being physically isolated [12].

Adolescents actively seeking help or social support online for their suicidal ideation might be more distressed than those who are able to seek help in the offline world, or those who do not seek help at all [13]. In this context, social media have been identified as potential avenues for suicide prevention interventions that can extend to otherwise hard-to-reach or underserved populations [14]. On the other hand, while the internet offers low-threshold access to social support and preventive interventions, it is also a source for potentially social contagious or harmful content such as unfiltered information on suicide methods [15].

Adolescents seeking for help or support online can turn to suicidal ideation support communities (SISC), which exist in the form of moderated or unmoderated forums and communities on self-hosted websites or as part of social media websites. SISCs can have both positive and negative impacts on young individuals [16]. When asking SISC members about their reasons for joining a SISC they mention meeting others with similar problems, receiving social support, and reducing social isolation [17]. Negative influences of SISCs extend to finding information on suicide methods or similar others to become suicide partners or form a suicide pact [18]. Being active in an SISC has also been associated with increases in suicidal ideation [19].

The manner in which communication of adolescents active in SISCs has changed since the outbreak of the COVID-19 pandemic could give valuable insights into how they are experiencing the pandemic and whether their level of suicidal ideation has changed during this time. But this area has so far received little attention in the research literature. One study found that adolescents’ participation in a SISC fluctuated highly while the level of negative emotions stayed relatively stable [20]. Another study found that posts in a SISC contained more references to loneliness and self-harm and got longer since the outbreak of the COVID-19 pandemic, however the authors did not specifically focus on adolescent users [21]. In the present study, we sought to investigate how the communication of adolescents with suicidal ideation has changed since the outbreak and worldwide spread of the COVID-19 pandemic by analyzing how they express themselves, what they write, and how active they are in a SISC.

The study of the language that individuals use when speaking or writing (sometimes referred to as linguistic style or lexical style analysis) has found numerous applications in research on suicidal behaviors—an individual’s language can serve as a proxy and provide valuable and unobtrusive insights into psychological processes in situations where individuals are not willing or able to express or report their emotions, thoughts or behaviors accurately [22]. In the area of suicide research, studies have used language analysis methods on suicide notes [23], Twitter posts of individuals with suicidal ideation [24], or artistic works such as songs or poems by artists that have committed suicide [25, 26] among others. Language analyses of Twitter posts found increased expressions of suicidal ideation in the first months of the COVID-19 pandemic relative to the same time period in 2019 [27]. Similarly, one aim of our study was to analyze the language style of posts and comments in a SISC during the time periods before and after the worldwide spread of the pandemic.

The language style, i.e., the way how individuals express themselves using specific words or groups of words, is one area of research on the role of language in suicidal ideation. This research has used language style as a proxy for understanding how suicidal individuals feel, think or behave. Researchers have used specific groups of words, such as negative emotion words or words related with death and dying, as markers for hopelessness and social disengagement, both of which are considered risk factors for suicidal ideation [25, 26]. This is based on the idea that hopelessness would express itself in writing through pervasive negative emotions such as sadness and anger as well as a preoccupation with death. Consequently, word categories of well-validated programs such as the Linguistic Inquiry and Word Count [28] that assess positive and negative emotion word use have been proposed as linguistic markers for hopelessness [25, 26]. For instance, in a study using patient language to identify suicide risk, decreased use of negative emotion words and an increased use of positive emotion words was associated with a decrease in suicidal ideation [29]. The opposite language pattern—an increase of negative and a decrease in positive emotion words—was found to predict increases participation in mental health communities such as SISCs [30]. In another study, the use of death-related words, which was proposed as an additional marker of hopelessness, differentiated between artists that had committed suicide and those that had not [25, 26]. Adolescents with suicidal ideation used more negative emotion words and death-related words as well as fewer positive emotion words in spoken interviews than controls [31]. In a recent study, the use of negative emotions as well as death-related words was higher in suicide-related Twitter posts than in non-suicide-related posts [24].

The role of social disengagement in the development of suicidal ideation and behaviors goes back to Durkheim’s famous study on suicide [32]. As many individuals found themselves physically isolated in the current pandemic, they might experience social disengagement or social isolation which, in turn, could increase their risk for suicidal ideation and behaviors. Accordingly, social disengagement was a major contributing factor to a rise in suicide rates during the SARS epidemic in Hong Kong in 2013 [33]. Research suggests that an increase in self-related words such as 1st person singular pronouns and a decrease of references to others, or words that refer to social processes, serve as language proxies for greater social disengagement [25, 26]. The underlying assumption is that individuals would detach from social life and become increasingly self-focused which would be mirrored in their language as fewer social references and higher self-focus. The use of self-referencing words, i.e., 1st person singular pronouns, in online writing as well as in interviews were found to differ between adolescents with suicidal ideation and non-suicidal individuals [31, 34]. Increases in 1st person singular pronouns and a decrease of other pronouns also predicted the future participation in an SISC among users of mental health communities, which was interpreted as a potential shift towards suicidal ideation in these users [35]. In a longitudinal study on participation in mental health communities, self-preoccupation also differentiated between users who increased their activity in SISCs and other mental health communities compared to less frequent users [30]. Besides signifying social disengagement, self-related words also predicted slower recovery from suicidal ideation and suicide risk in suicidal outpatients [36].

The present study also aims at providing a deeper understanding about the most prevalent themes and topics in SISC by exploring what individuals are discussing and how these topics have changed during the COVID-19 pandemic. Researchers seeking to identify discussion topics in a large collection of texts such as all posts and comments by adolescents in a SISC may use topic modelling with Latent Dirichlet Allocation (LDA), a type of unsupervised, bottom-up machine learning [37] for this task. Topic Modelling with LDA has been applied to online communities related to eating disorders [38], depression [39] as well as SISC [40]. The latter study showed that topic modeling of adolescents’ suicide-related posts produces topics that are comparable to those created by human coders. We expected that changes in prevalence of topics in posts and comments of adolescent users would reflect the changing importance of these topics in the COVID-19 pandemic.

As a final measure for changes in the COVID-19 pandemic we explored the user activity of adolescents in the SISC. Activity can be measured in a number of different ways, e.g., by counting the number of posts and comments made, the number of posts and comments in a specified time period or by measuring the length of text users write. A number of studies have explored how user activity relates to distress and suicidal ideation. Individuals that increased their activity in mental health communities also showed more markers for negative emotions and social isolation in their language [30]. How much users write, i.e., their word count, has been found to be associated with suicidal ideation [24, 41]. In earlier work, however, greater activity in the form of the number of messages sent to other users and messages received from other users predicted lower emotional distress a month later [42]. User activity in a SISC can also change as a reaction to real life events, as one study demonstrated with higher number of posts following suicides of celebrities [43]. Since the outbreak of the COVID-19 pandemic, adolescents with mental health concerns have increased their activity in non-mental health related community [20]. To investigate whether a similar increase in activity occurred in a SISC, we compared the user activity of adolescents in a SISC during the time period since the outbreak of the worldwide COVID-19 pandemic with their user activity before that.

In sum, the current study raises three research questions regarding the communication of adolescents in a suicide ideation social community during the COVID-19 pandemic: have linguistic markers for hopelessness and disengagement in posts and comments changed since the outbreak of the pandemic? How has the content of posts and comments changed since the outbreak of the pandemic? Has the user activity of adolescents changed since the outbreak of the pandemic?

## Methods

### Study design

In this retrospective observational study, we investigated the SISC *r/SuicideWatch* on the social media website reddit.com. *Reddit* is an international social media website that consists of thousands of communities, so called subreddits, dedicated to different thematic areas such as sports, movies or politics but also to mental health issues like depression or suicidal ideation. *Reddit* users can write posts and comment on other users’ posts in these communities or create their own community around a chosen theme. Communities are moderated by volunteers who create rules on what kind of content is allowed in their community. The SISC *r/SuicideWatch*, which is one of the largest social network communities in this context, was created in 2008 and currently has around 330,000 members [44]. It has been the subject of numerous studies investigating topics such as daily and weekly variations in number of posts and comments [45], changes in activity following suicides by celebrities [43], or users transitioning from mental health issues to suicidal ideation [35].

We investigated whether effects of the COVID-19 pandemic can be detected in linguistic markers for suicidal ideation (language style), content, and user activity in posts and comments by adolescent users of the SISC. We conducted within-person comparisons of these measures between the time period before the worldwide spread of the COVID-19 pandemic and the time period since then. We chose March 11, 2020, as the cutoff date for the time periods as COVID-19 was declared a worldwide pandemic by the World Health Organization on this day [46]. Every post and comment made before this cutoff was considered as “pre-pandemic” while everything posted after March 11, 2020, was treated as being in the “mid-pandemic” time period. Only users that wrote at least one post or comment during both time periods were included in the study. To limit our analysis to adolescent users, we only included users who had actively participated in *r/teenagers* [47], a *Reddit* community for teenagers, in the past following a method recently employed by another study [20].

### Data collection

We collected posts and comments from *r/SuicideWatch* over a period from April 2020 to September 2021 through the *Reddit* application programming interface (API) using the R package redditoR [48]. For all active users during this time period, we also collected the posts and comments they had made in the SISC before April 2020. There were 15,800 users that had participated in *r/SuicideWatch* during both, the pre- and the mid-pandemic time period. Of these users, 2977 individuals had also participated in *r/teenagers* in the past. We excluded 33 users because they had participated in *r/teenagers* for longer than seven years, and thus, would not be considered teenagers anymore. Therefore, posts and comments by 2944 adolescent SISC users were considered for inclusion in the present study.

### Data preparation

Posts and comments in a language other than English (*n* = 27) were identified using the R packages cld2 [49] and cld3 [50] and removed from the sample. We removed double postings, i.e., multiple posts (*n* = 17) or comments (*n* = 54) with the same content made in a short period of time, retaining only the first post or comment. Further posts were removed because their content had been deleted by the user or by a community moderator (*n* = 239). Finally, we preprocessed posts and comments by removing all HTML code, Unicode characters, and emojis. Due to these preparation steps, 21 users had to be excluded from the sample because they did not fulfill the inclusion criteria anymore, e.g., they did not have posts and comments in both time periods. For the text analyses, we concatenated all posts and comments by each user in each time period into one text document.

### Data analysis

We used the Linguistic Inquiry and Word Count (LIWC) 2015 text analysis program [28] to assess language style markers for hopelessness and social disengagement. Based on its internal dictionary, LIWC groups words with similar meanings into language categories such as Negative Emotions, Positive Emotions, or Death. The program’s algorithm counts the number of times each category word appears in a given text and calculates the number of category words as a percentage of all words in the text as a category score. For this study, we selected LIWC categories that had been used in previous studies to capture the linguistic expression of hopelessness (*Positive Emotions*, *Negative Emotions*, and *Death*) and social disengagement (*Social Processes*, *1st Person Singular Pronouns*, *1st Person Plural Pronouns*, and *2nd Person Pronouns*) [25, 26]. Example words for these LIWC categories can be found in Table 1. Because dictionary-based word count algorithms are sensitive to misspellings, typos or other problems, such as non-English language use, we checked the results from the LIWC program for documents that were not well-covered by its dictionary and retained only documents with a good text quality. To this end, we used one of LIWC’s summary variables (*Dictionary Words*) which calculates the percentage of each document’s words that have been captured by the program’s dictionary. We excluded a given document if less than 70% of its words were covered by LIWC dictionary as the minimum requirement for inclusion in this study to ensure good reliability of the results [38]. Only fifty-one users with documents below this threshold had to be excluded from the analyses.

**Table 1:** Prevalences of linguistic markers for hopelessness and social disengagement in adolescent users’ posts and comments in a suicidal ideation self-help community on the social media website *Reddit* in the pre- and mid-pandemic time periods

Linguistic category	Example words	Pre-pandemic median (IQR)	Mid-pandemic median (IQR)	*Z*	*p*
Linguistic markers for hopelessness					
Positive emotion	Love, nice, sweet, happy	3.62 [2.32–5.26]	3.5 [2.22–5.08]	− 2.96	< .01
Negative emotion	Hurt, ugly, nasty, sad, angry	3.5 [2.13–4.95]	3.85 [2.37–5.36]	5.21	< .01
Death	Dying, bury, coffin, kill	0.59 [0–1.29]	0.58 [0–1.37]	0.519	0.6
Linguistic markers for social disengagement					
Social processes	Mate, talk, they	11.18 [7.35–15.79]	10.21 [6.41–14.79]	− 6.19	< .01
2nd person pronouns	You, your, thou	3.15 [0.5–6.9]	2.08 [0.27–6.18]	− 6.57	< .01
1st person singular pronouns	I, me, mine	9.41 [5.21–12.93]	9.67 [5.05–13.18]	0.305	0.76
1st person plural pronouns	We, us, our	0 [0–0.35]	0 [0–0.32]	− 0.876	0.38

Example words for LIWC categories are taken from the LIWC manual [28]. Pre-pandemic time period: posts and comments until March 11, 2020; mid-pandemic time period: posts and comments between March 11, 2020 and September 2021

*IQR* interquartile range.

Changes in prevalence between pre- and mid-pandemic time periods were tested with Wilcox signed-rank tests

The R package stm [51] was used to estimate a structural topic model with LDA. For the topic modelling, additional preprocessing of the text documents consisted of the removal of numbers, punctuation marks, and stopwords, i.e., words that are very common but do not carry a lot of meaning such as “are” or “it”. Additionally, all words were turned in to their word stem, e.g., the words “hopelessness” and “suicidal” would be turned into “hopeless” and “suicid”. After these preprocessing steps, 10 users were removed from the sample because their posts and comments in one time period were rendered blank by the preprocessing steps. We removed all words that appeared in less than three different documents to speed up computation. This led to the exclusion of two users whose text documents consisted of very rare words (e.g., a user whose only pre-pandemic comment consisted of the word “Uhhhhhh”).

We included the time period (pre-pandemic/mid-pandemic) as a covariate in our model allowing prevalence of topics to vary according to whether posts and comments had been written before or after March 11. The number of topics, *K*, is a model parameter that has to be carefully chosen and evaluated by researchers. Initially, we estimated a number of models with *K* = 9–30. The evaluation of models was based on the statistical measures exclusivity [52] and semantic coherence [53], as well as the interpretability of topics through manual inspection of their most characteristic words and texts. A model with *K* = 21 topics was chosen as the final model because it had high values in both exclusivity and semantic coherence, and most of its topics were intelligible. The remaining topics were labeled by the first author after manual inspection of characteristic words and texts. Characteristic words were selected according to the FREX metric, which calculates the harmonic mean of the frequency of a word in a topic and its exclusivity for the topic [54]. We present selected topics with their topic labels and their ten most characteristic words in Table 2. Results for the other topics in our final model can be found in Online Appendix 1.

**Table 2:** Prevalences of topics in adolescent users’ posts and comments in a suicidal ideation self-help community on the social media website *Reddit* in the pre- and mid-pandemic Time periods

Topic	FREX words	Pre-Pandemic Median (IQR)	Mid-Pandemic Median (IQR)	*Z*	*p*
Suicide Methods	Car rope gun smoke knife walk neck floor belt wall	0.04 [0.02–0.07]	0.04 [0.02—0.08]	5.87	< .01
Suicide Attempts	Pill overdos mg attempt numb week wake od tonight ward	0.05 [0.03–0.09]	0.05 [0.03—0.09]	1.62	0.11
Social Support	Help can listen mayb hear understand trust glad sorri someon	0.05 [0.03–0.06]	0.04 [0.03—0.06]	− 11.7	< .01
Reaching Out	Hey pleas pm dm chat hi ok okay alright thank	0.05 [0.02–0.1]	0.03 [0.02—0.07]	− 13.8	< .01
Hopelessness	Fuck pop nobodi anymor tire hate shit noth wast pathet	0.07 [0.04–0.13]	0.09 [0.05—0.16]	10	< .01

Characteristic words for topics were determined using the FREX metric: Harmonic mean of frequency and exclusivity of a word in a topic [54]. Pre-pandemic time period: posts and comments until March 11, 2020; mid-pandemic time period: posts and comments between March 11, 2020, and September 2021

*IQR* interquartile range. Changes in prevalence between pre- and mid-pandemic time periods were tested with Wilcox signed-rank tests. Displayed in the table are 5 out of 21 topics. The remaining topics, their labels, prevalence, and characteristic words can be found in Supplement 1

The user activity of SISC users during both time periods was assessed based on an established set of measures: The number of posts or comments written [20]; ratio of posts to comments [55]; number of words (sum of words in posts and comments) and the number of words per post/comment [35, 43]; and the number of posts and comments per week [56]. The latter two measures were calculated by dividing the number of posts or comments by the total participation time in seconds and subsequently multiplied by the number of seconds in a week. The two measures were included since users participated in the SISC for time periods of varying lengths.

The final sample of this study consisted of *N* = 2862 adolescent users who wrote a total of 3707 posts and 29,169 comments before March 11, and 4268 posts, and 21,950 comments after March 11, 2020. Differences in LIWC categories, topic prevalence, and user activity measures between the pre- and mid-pandemic time periods were tested using Wilcoxon Signed-Rank Tests as most of the measures were not normally distributed. The *Z* and *p* values for the three different types of measures are presented in Tables 1, 2 and 3 respectively. Additionally, we calculated the relative change of the language style categories and LDA topics in the pre- to the mid-pandemic time period, with numbers from the pre-pandemic time period as a baseline.

**Table 3:** Measures of activity of adolescent users in a suicidal ideation self-help community on the social media website *Reddit* in the pre- and mid-pandemic time periods

Activity measure	*n* Users	Pre-pandemic median (IQR)	Mid-pandemic median (IQR)	*Z*	*p*
N posts	921	1 [1–3]	2 [1–3]	− 0.305	0.76
N comments	2220	4 [2–11]	3 [1–7]	9.36	< .01
Ratio of posts to comments	534	0.33 [0.17–0.75]	0.5 [0.25–1]	− 7.49	< .01
N posts per week	630	0.19 [0.07–0.67]	0.17 [0.07–0.63]	0.035	0.97
N comments per week	1505	0.99 [0.3–6.68]	0.54 [0.16–3.94]	5.44	< .01
N words	2862	195 [60–533.75]	177 [62–449.5]	3.74	< .01
N words per post/comment	2862	40.18 [20–78.75]	45 [23.19–86]	− 4.64	< .01

Pre-pandemic time period: posts and comments until March 11, 2020; mid-pandemic time period: posts and comments between March 11, 2020, and September 2021

*IQR* interquartile range. Changes in activity measures between pre- and mid-pandemic time periods were tested with Wilcox Signed-Rank Tests. *n* users: the number of users that had values for the activity measure in both time periods and with whose data the analysis of the activity measure was conducted

## Results

### Linguistic markers for hopelessness

**Fig. 1:**
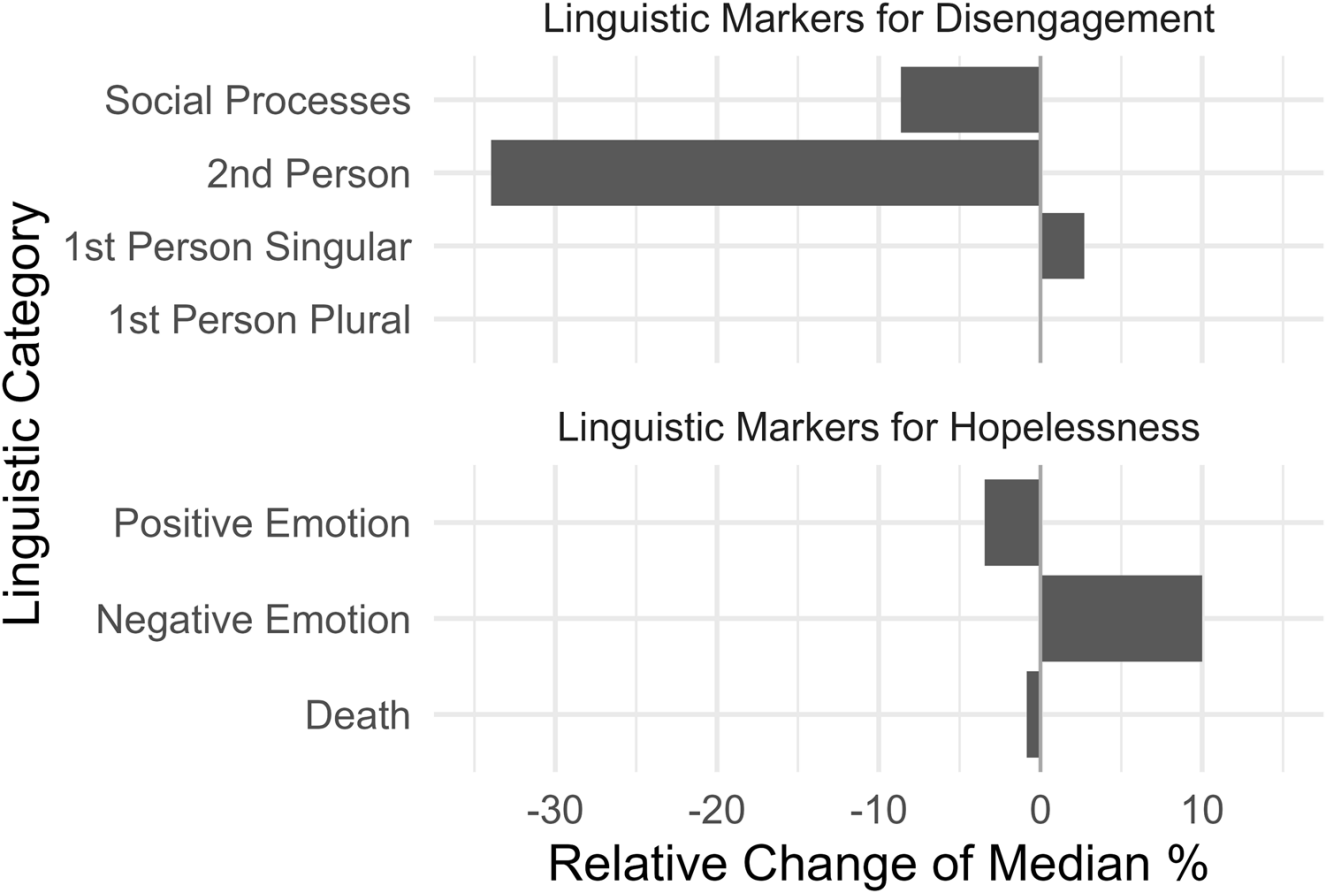
Relative change of medians of linguistic markers for social disengagement and hopelessness in adolescent users’ posts and comments in a suicidal ideation self-help community on the social media website *Reddit* between the pre- and mid-pandemic time

Two linguistic markers for hopelessness changed significantly among adolescent users of the SISC *r/SuicideWatch* since the beginning of the COVID-19 pandemic (see Table 1 and Fig. 1). We observed a relative decrease of − 3.45% in the percentage of* Positive Emotion *words in mid-pandemic posts and comments. Additionally, users wrote significantly more words assigned to the* Negative Emotions *category during the COVID-19 pandemic (+ 10.00%). There was no significant change in the number of times users employed words associated with Death.

### Linguistic markers for social disengagement

Since the onset of the pandemic, two of the four linguistic markers for social disengagement have decreased: The proportion of words categorized under the *Social Processes* category (− 8.63%) and *2nd Person Pronouns* (− 33.97%) were significantly reduced. The use of first-person pronouns, both singular (+ 2.71%) and plural (− 0.00%), did not show significant changes between the two time periods.

### Topics

**Fig. 2:**
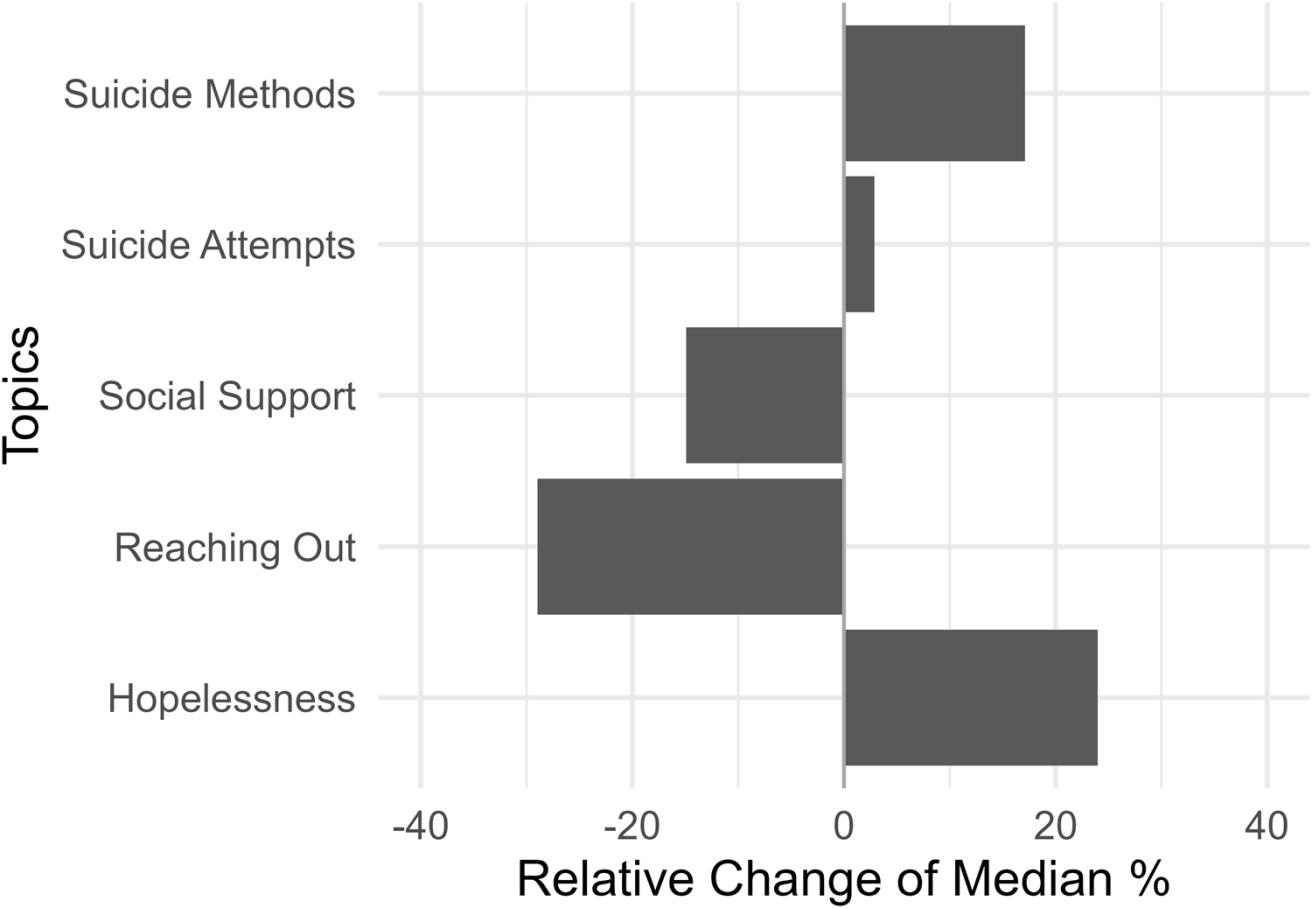
Relative change of medians of topic prevalence in adolescent users’ posts and comments in a suicidal ideation self-help community on the social media website *Reddit* between the pre- and mid-pandemic time periods

We also observed a significant change in prevalence that was labeled as *Hopelessness*, which showed a relative increase of more than 20% in the mid-pandemic time period compared to the pre-pandemic time period (see Table 2 and Fig. 2). The prevalence of the topics *Social Support* and *Reaching Out* both decreased significantly with relative changes of -14.91% and -28.97% respectively. Discussions around *Suicide Methods* became more prevalent during the pandemic with a relative increase of 17.11%, while the prevalence of the topic *Suicide Attempts* did not change significantly.

### User activity

The average number of posts a user wrote in both time periods did not change (see Table 3). There was a significant decrease in the average number of comments per users in the mid-pandemic time period leading to a significantly higher posts-to-comments ratio during that time. The average number of comments per week was significantly lower in the mid-pandemic time period, whereas the average number of posts per week did not change significantly. There was a significant decrease in the total number of words per user and a significant increase of words per post/comment in the mid-pandemic time period.

## Discussion

In the present study, we investigated communication patterns by adolescent users in one of the largest SISC on the internet, *r/SuicideWatch* on the social media website *Reddit*. Specifically, we tested whether language style, content, and activity changed from before to after the worldwide spread of the COVID-19 pandemic. We selected adolescent users who were active in this community before the pandemic, and thus, were assumed to have some level of suicidal ideation prior to its worldwide spread. We observed significant changes in the mid-pandemic time period in word groups that have been proposed as linguistic markers for social disengagement and hopelessness and have been linked in previous research to suicidal ideation [25, 26, 34].

Additionally, linguistic features associated with sharing of social support and initiating social interactions between users, such as the topics *Social Support *and* Reaching Out*, have decreased. Furthermore, we found that the topic* Suicide Attempts* became more central after the outbreak of the COVID-19 pandemic. These findings are concerning as they point to a potential worsening in the expression of suicidal ideation, and might be associated with an increase in suicidal behaviors. A similar decrease of social support was documented in an online eating disorder community during the initial onset of the COVID-19 pandemic [57].

Our findings show a reduction in interactivity which could be an indicator for greater social disengagement among adolescent social media users. During the pandemic, adolescent users were addressing other SISC users less often and used fewer words related to social processes. Lowered interactivity as assessed through the expression of* 2nd Person pronouns *were also found for users shifting from other mental health communities on *Reddit* to *r/SuicideWatch* [35]. The lessened interactivity was also illustrated by the decrease of the overall number of comments a user wrote since the spread of the pandemic.

We also observed a significant decrease of the topics *Social Support* and *Reaching Out*. This might be related to the lower number of comments as support and outreach is most often expressed in comments to other users. The decrease of these topics is noteworthy as social support by peers has been shown to be an important factor for the development of suicidal ideation in users of mental health communities [58], while in another study it was mentioned as one of the main reasons for participating in SISCs [16]. At the same time, lockdowns and school closures prevented adolescents from socializing with offline peers and from accessing their normal social support networks [9].

The unchanged use of *1st Person Singular Pronouns*, which is another linguistic marker for social disengagement, indicates that adolescents did not become more self-focused which has been discussed in the literature as a generic indicator for psychological distress [59, 60]. It should be noted, however, that the level of self-focus was high in our sample already which is to be expected in a sample of individuals with mental health issues or suicidal ideation [34].

The increased use of *Negative Emotions* words and decreased use of *Positive Emotions* words indicated a potential rise of hopelessness among adolescent users during the COVID-19 pandemic when compared to the time before the pandemic. Interestingly, we did not observe a significant change in the LIWC category* Death *between the two time periods. This might be explained by the fact that, again, the prevalence of death-related words in the pre-pandemic time period was comparably high already (Median = 0.59, IQR = [0–1.29]), also when compared to other suicide related corpora [25, 26]. Finally, these results are further complemented by a significant increase in the prevalence of the topic* Hopelessness* from our topic model.

Furthermore, discussions around suicide methods seem to have increased in the SISC since the spread of the COVID-19 pandemic as an increase of the topic* Suicide Methods* in our study indicates. This is worrying as theories in the ideation-to-action framework such as the Three-Step Theory [61] would indicate that these developments might lead adolescent users from experiencing suicidal ideation to engaging in suicidal behaviors in real life. The theories see greater knowledge about suicide methods and access to suicide methods, as might be discussed in the topic *Suicide Methods*, as increasing a person’s capacity to engage in suicidal behaviors. Discussing suicide methods in an SISC might also increase the risk of uptake or imitation by other users in a similar way as discussions of methods for engaging in self-destructive behaviors or hiding them from others in online communities for self-harm or eating disorders [62].We did not observe significant changes in the topic *Suicide Attempts*. This is noteworthy as reading about suicide attempts or completed suicides in online communities has been linked to increases in suicidal ideation among adolescents [19].

Finally, we investigated how adolescent users’ activity has changed from the pre-pandemic to the mid-pandemic time period. Based on the indicators used in our study, we could not find a general increase in adolescents’ activity in the SISC since the start of the pandemic, unlike the findings of adolescents’ increased activity in non-mental health-related communities [20]. Instead, users’ overall activity in the form of posts and comments decreased. The number of comments decreased while the number of posts increased, leading to a significant shift in the ratio of posts to comments towards a higher number of posts to comments. The number of posts and comments per week, however, decreased in the mid-pandemic period, but only change in the comments per week measure was significant with the median number of comments per week reduced by almost half. The overall word count for each user decreased significantly, while the number of words users wrote per post or comment increased significantly. The higher word count per post or comment could point to increased self-disclosure and, potentially, development of suicidal ideation [35]. Taken together, these results suggest the teenagers´ higher need to express or communicate their mental health issues and suicidal ideation in the form of more and longer posts during the COVID-19 pandemic while the lower number of comments indicates fewer interactions and lower engagement with other users.

### Limitations and strengths

A first limitation is concerned with how the sample of posts and comments were split into pre- and mid-pandemic time periods. All posts and comments made since March 2020 were treated as one time period, thereby obscuring possible different waves of the pandemic. However, collapsing data taken from a longer time frame since the onset of the pandemic can also be considered a strength of the study as it allowed us to reach more valid conclusions about our study sample that go beyond short-term trends (see [63]).

A second limitation is that we only included adolescent users that had already been active in the SISC before the pandemic, and thus, were presumed to have suffered from suicidal ideation for a longer period of time already. While this approach increased the validity of our study, and allowed us to analyze within-person changes, future studies should investigate adolescents that joined this community during the COVID-19 pandemic. Our study does not allow to draw conclusions for that group.

Since the present study was observational, we cannot rule out that the observed changes are caused by factors other than the COVID-19 pandemic such as changes in personal circumstances, a gradual increase in suicidal ideation or a change in how active adolescents were on *Reddit* in general.

As we based our observational study exclusively on the writing and activity of users in a SISC, we have no data for independent validation of our results. While language style and content can serve as a proxy for thoughts, emotions or behaviors of the writer, there is no direct causal connection between these concepts and written language. Although the language categories used in our study have repeatedly been proposed as markers for hopelessness and social disengagement, further validation is warranted. Nevertheless, a growing body of research indicates that there is a substantial link of these word groups to suicidal ideation [24–26, 29, 30, 34, 35]. Furthermore, we cannot infer that the changes in linguistic features and topics of posts and comments in the SISC translate to changes in suicidal thoughts and behaviors in real life. This is an issue that most studies on suicidality on the internet and in online communities face as only few have been able to link language analyses to other data sources for further validation (for an example see [64]).

We cannot be certain whether all users in our sample were adolescents, and whether all were suffering from suicidal ideation. Adults could be active in the *r/teenagers* community and users could post and comment in the SISC for reasons other than their own suicidal ideation or behaviors, e.g., wanting to help and support others. Furthermore, some adolescents with suicidal ideation might have been excluded from our sample because they created separate “throwaway” accounts for their activity in the SISC [65]. However, the method for selecting our sample was economical and has been used in studies before [20, 56].

A final limitation is that, due to the exploratory nature, we have not controlled our analyses for multiple comparisons made in our study.

### Lessons learned and consequences for the future

The changes in linguistic markers for suicidal ideation and topics we uncovered in our study point to an increase in suicidal ideation as well as the capacity for suicide since the worldwide spread of the COVID-19 pandemic. Results on language style and content were concordant, pinpointing the validity of the analyses. These results are concerning, as they could indicate a potential rise in the likelihood to engage in suicidal behaviors among adolescents with prior suicidal ideation during the COVID-19 pandemic. Our results are in line with recent epidemiological findings of increases in admissions of adolescents with suspected suicide attempts in pediatric hospitals [7, 8].

The study clearly demonstrates the value of psycholinguistic analyses in the research on suicidal ideation of adolescents. Such analyses could help to uncover trends in the level of suicidal ideation in specific groups and in response to specific events such as the COVID-19 pandemic or celebrity suicides [43]. On an individual level, linguistic markers could be used to identify SISC users that are at risk for worsening suicidal ideation or suicidal behaviors and offer them timely preventive interventions. Yet, future studies should strive to validate linguistic markers in posts and comments in SISC with external criteria such as surveys, interviews with users, or registries.

## Supplementary Information

Below is the link to the electronic supplementary material.

Supplementary file1 (DOCX 61 KB)
