# Supplementary material for: Psycholinguistic changes in the communication of adolescent users in a suicidal ideation online community during the COVID-19 pandemic
Source: Eur Child Adolesc Psychiatry. 2022 Aug 26;32(6):975–85. doi: 10.1007/s00787-022-02067-7 (PMC9415261; doi:10.1007/s00787-022-02067-7)

Supplement

Table S1. Remaining Topics: Prevalences in Adolescent Users’ Posts and Comments in a Suicidal Ideation Self-Help Community on the Social Media Website *Reddit* in the Pre- and Mid-Pandemic Time Periods

| Topic | FREX Words | Pre-Pandemic Median (IQR) | Mid-Pandemic Median (IQR) | *Z* | *p* |
| --- | --- | --- | --- | --- | --- |
| Leisure | game movi play tv sex women anim fish gym favourit | 0.02 [0.01 - 0.05] | 0.02 [0.01 - 0.05] | 1.57 | 0.12 |
| Stories | anguish particular hello necessari inadequ familiar endur despair torment favor | 0 [0 - 0.01] | 0 [0 - 0.01] | 6.51 | < .01 |
| Distractions | tri like want enjoy nice thing wrong mind idea burden | 0.05 [0.04 - 0.06] | 0.05 [0.04 - 0.06] | -8.57 | < .01 |
| Ethics | rule platitud ultim etern concept exist belief conscious content creat | 0.01 [0.01 - 0.03] | 0.02 [0.01 - 0.05] | 13.4 | < .01 |
| Education | school grade dad class mom father colleg sister yell brother | 0.03 [0.02 - 0.06] | 0.03 [0.02 - 0.05] | -3.03 | < .01 |
| No one cares | care one eat weight calori amount bother delay exampl mention | 0.01 [0.01 - 0.01] | 0.01 [0.01 - 0.01] | -10.3 | < .01 |
| Mental Health Treatment | psychiatrist therapist therapi medic resourc doctor compass awhil connect hard | 0.01 [0.01 - 0.02] | 0.01 [0.01 - 0.02] | -0.484 | 0.63 |
| Positivity | strong beauti love worth fight us stronger tough op happi | 0.04 [0.03 - 0.07] | 0.04 [0.02 - 0.07] | -2.77 | 0.01 |
| Future Focus | may step easi goal focus truli futur choic mistak path | 0.04 [0.02 - 0.06] | 0.04 [0.02 - 0.06] | -4.93 | < .01 |
| Emotions | feel lot hope need good believ struggl kind word person | 0.04 [0.03 - 0.05] | 0.04 [0.03 - 0.05] | -4.59 | < .01 |
| Reddit Community | sub comment subreddit assum post internet opinion situat genuin commit | 0.04 [0.03 - 0.07] | 0.04 [0.02 - 0.06] | -2.15 | 0.03 |
| Making a Change | right get see peopl tell depress think hurt write bad | 0.04 [0.03 - 0.05] | 0.04 [0.03 - 0.05] | -7.04 | < .01 |
| Stressors | mental stress now deal work job enough health issu keep | 0.03 [0.02 - 0.04] | 0.03 [0.03 - 0.04] | 10.5 | < .01 |
| Human Connection | thought much realli know long though part away way mine | 0.04 [0.03 - 0.04] | 0.04 [0.03 - 0.04] | -0.946 | 0.34 |
| Reasons to Live/Die | guess probabl honest actual pretti just reason stuff yeah weird | 0.07 [0.05 - 0.08] | 0.06 [0.05 - 0.08] | -7.91 | < .01 |

Note. Characteristic words for topics were determined using the FREX metric: Harmonic mean of frequency and exclusivity of a word in a topic [54]. Pre-pandemic time period: posts and comments until March 11, 2020; mid-pandemic time period: posts and comments between March 11,2020 and September 2021. IQR = Interquartile Range. Changes in prevalence between pre- and mid-pandemic time periods were tested with Wilcox Signed-Rank Tests.

Figure S1. Relative Change of Medians of Topic Prevalence in Adolescent Users’ Posts and Comments in a Suicidal Ideation Self-Help Community on the Social Media Website *Reddit* between the Pre- and Mid-Pandemic Time Periods


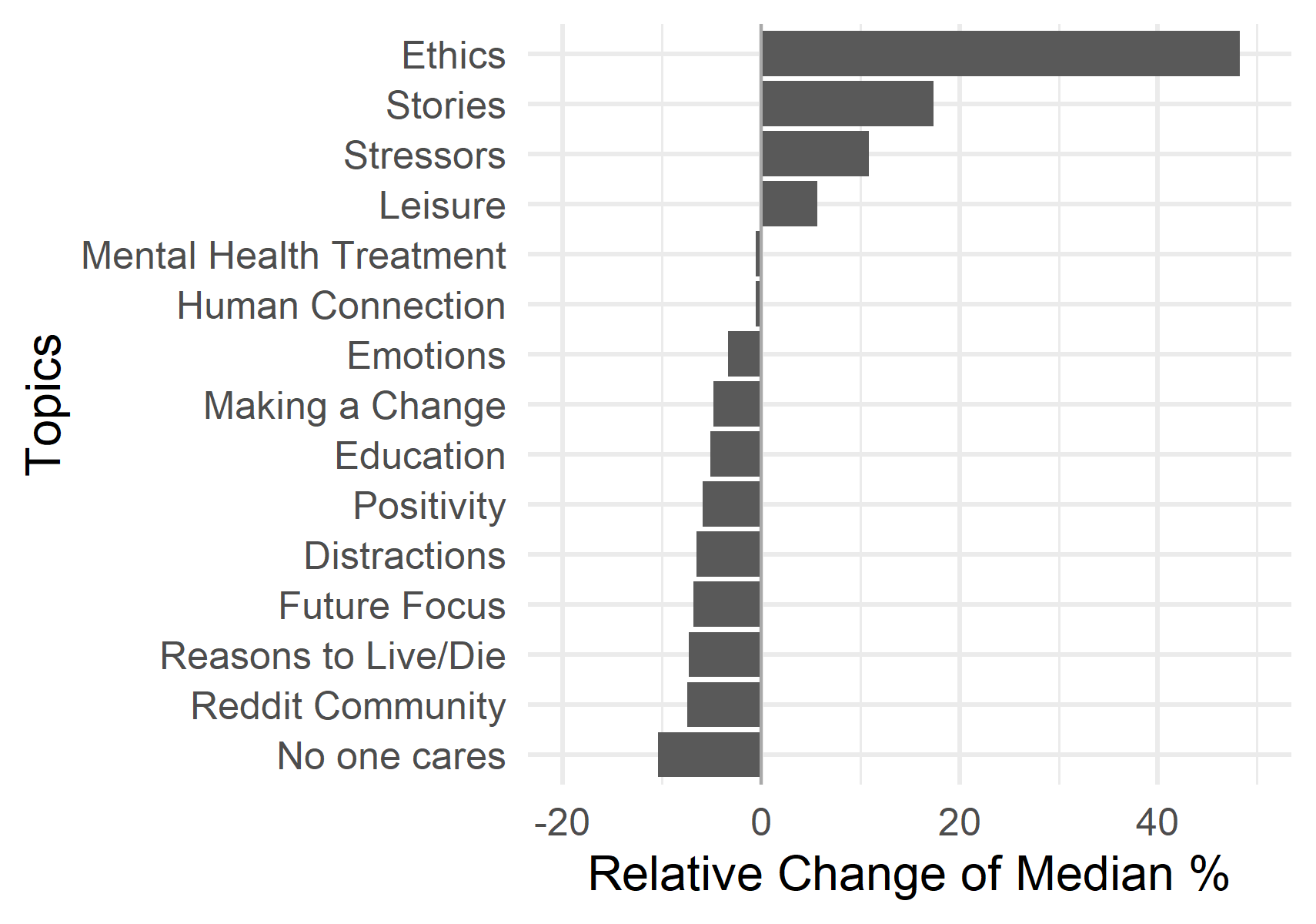

Supplement: Supplementary file 1 — Supplementary file1 (DOCX 61 KB) [file 787_2022_2067_MOESM1_ESM.docx]
